# Immune Response in Vitamin D Deficient Metastatic Colorectal Cancer Patients: A Player That Should Be Considered for Targeted Vitamin D Supplementation

**DOI:** 10.3390/cancers14112594

**Published:** 2022-05-24

**Authors:** Cristina Morelli, Michela Rofei, Silvia Riondino, Daniela Fraboni, Francesco Torino, Augusto Orlandi, Manfredi Tesauro, Giovanna Del Vecchio Blanco, Massimo Federici, Hendrik-Tobias Arkenau, Vincenzo Formica, Mario Roselli

**Affiliations:** 1 Medical Oncology Unit, Department of Systems Medicine, Tor Vergata University Hospital, 00133 Rome, Italy; cristina.morelli89@gmail.com (C.M.); rofei.michela@gmail.com (M.R.); silviariondino2@gmail.com (S.R.); torino@med.uniroma2.it (F.T.); mario.roselli@uniroma2.it (M.R.); 2 Department of Biomedicine and Prevention, University of Rome “Tor Vergata”, 00133 Rome, Italy; danielafraboni@virgilio.it; 3 Anatomic Pathology, Department of Biomedicine and Prevention, University of Rome “Tor Vergata”, 00133 Rome, Italy; orlandi@uniroma2.it; 4 Department of Internal Medicine, University of Rome “Tor Vergata”, 00133 Rome, Italy; mtesauro@tiscali.it; 5 Gastroenterology Unit, Department of Systems Medicine, University of Rome “Tor Vergata”, 00133 Rome, Italy; giovanna.del.vecchio.blanco@uniroma2.it; 6 Department of Systems Medicine, University of Rome “Tor Vergata”, 00133 Rome, Italy; federicm@uniroma2.it; 7 Sarah Cannon Research Institute, Cancer Institute, University College London, London W1G 6AD, UK; tobias.arkenau@hcahealthcare.co.uk

**Keywords:** colorectal cancer, targeted therapies, predictive markers

## Abstract

**Simple Summary:**

In this study, we investigated the role of vitamin D levels and the impact on immune response in patients with mCRC and propose a vitamin D cut-off level. Among several hematological, biochemical and immunological variables, we identified the neutrophils-to-lymphocytes ratio, CD4+ T lymphocytes and B lymphocytes as being closely related to vitamin D status with an impact on survival.

**Abstract:**

Background: Vitamin D deficiency is a poor prognostic factor in metastatic colorectal cancer (mCRC); however, targeted supplementation trials have so far yielded limited results. We investigated clinical-laboratory parameters influencing vitamin D deficiency, with a particular focus on immune response, and the effect on survival. These parameters could help optimize targeted supplementation therapy. Methods: Association of plasma 25-hydroxyvitamin D (25(OH])D) with overall survival (OS) was assessed with the Hazard Ratio Smoothed Curve with Restricted Cubic Splines (HRSC-RCS) and maximally selected rank statistics (MSRS) in mCRC patients who underwent first-line chemotherapy. Several hematobiochemical variables were evaluated as predictors of vitamin D deficiency by means of Least Absolute Shrinkage and Selection Operator (LASSO) analysis. In a patient subset, peripheral lymphocyte subpopulations were also analyzed. Results: One hundred thirty-three mCRC patients were included. The median(m) baseline 25(OH)D was 10.8 ng/mL (range 3–53.4). HRSC-RCS revealed a linear association between 25(OH)D and OS. MSRS found 10 ng/mL as the optimal 25(OH)D cut-off. The median OS for 25(OH)D < 10 (*n* = 60) vs. > 10 ng/mL (*n* = 73) was 12.3 and 24.5 months, respectively (*p* = 0.002). The LASSO analysis identified high neutrophil-to-lymphocyte ratio (NLR > 3.5) as the strongest predictor of vitamin D deficiency (Odds Ratio 3.35, *p* 0.0009). Moreover, patients with low 25(OH)D levels (< 10 ng/mL) and high NLR (>3.5) had the shortest survival and patients with 25(OH)D >10 ng/mL and NLR <3.5 had the longest: mOS 8.1 and 28.1 months, respectively, HR 3.40 (1.76–6.59), *p* 0.0004. Besides the significant difference in NLR between 25(OH)D < and > 10 ng/mL patients (mNLR 3.6 vs. 2.9, *p* 0.03), the lymphocyte subpopulation analysis revealed that vitamin D deficiency was associated with high T- CD4+ (*p* = 0.04) and low B (*p* = 0.03) lymphocyte frequency. Conclusions: NLR is a powerful predictor of Vitamin D deficiency and can further help in stratifying prognosis. Vitamin D deficiency was associated with significant variations in peripheral immune cells. We hypothesize that integrated targeted interventions to both vitamin D and immune system would improve the prognosis of mCRC patients.

## 1. Introduction

Colorectal cancer (CRC) is the second cause of cancer-related death in the United States and in Europe [1,2]. Around 20% of the cases present with stage IV disease at the diagnosis and this is associated with a 5-year survival rate lower than 15% [3]. Although about 20% of cases arises on a hereditary-family basis, more than 50% of the CRCs are caused by potentially modifiable risk factors, such as physical inactivity, cigarettes smoking, poor dietary calcium and fiber intake [4].

The correlation between vitamin D3 (cholecalciferol) and cancer has been extensively explored. Previous in vitro studies on several tumor types, including CRC, have shown that the vitamin D active form, 1α,25-dihydroxyvitamin D3 [1,25(OH)2D3] (calcitriol), is able to promote cell differentiation and inhibit tumor invasion and proliferation upon binding to its receptor VDR [5]. Furthermore, plasma concentration of 25-Hydroxyvitamin D3 (25(OH)D) has been found to correlate with risk of developing colorectal adenoma and adenocarcinoma and lower expression of vitamin D receptor (VDR) has been observed in colorectal adenocarcinoma cells as compared to adjacent normal tissue [6].

Besides a direct effect on cancer cells, vitamin D might also indirectly affect cancer growth by modulating innate and adaptive immunity. Previous genome-wide analyses have explored all the genes regulated by vitamin D in immune cells such as macrophages, monocytes, dendritic cells, B and T cells. Overall vitamin D target genes in these cells have anti-inflammatory and immune tolerance actions as well as anti-tumor immunity effects [7,8,9].

A protective role of vitamin D has been suggested for a number of pathological conditions other than cancer, such as cardiovascular, metabolic and immunologic diseases [10,11,12,13].

Despite the consistent evidence that low plasma 25(OH)D levels represent a risk factor for many diseases and illnesses, randomized trials investigating the preventive and curative role of targeted therapy with oral vitamin D3 administration have been largely disappointing [14,15,16] and doubts have been raised on the utility of the screening for vitamin D deficiency in adults [17].

In colorectal cancer, a correlation between low concentrations of circulating 25(OH)D and increased incidence of CRC has been found in many epidemiological studies [18], and a correlation with impaired prognosis has been demonstrated in patients with advanced CRC [19]. However, no specific cut-off value of 25(OH)D level has been identified to precisely predict risk or prognosis of CRC.

More recently, a randomized study comparing oral targeted therapy with vitamin D3 at two different doses (4000 vs. 400 UI/day) in combination with standard first-line chemotherapy in metastatic CRC (mCRC) found that higher dose vitamin D was associated with a non-significant improvement in progression free survival [15].

The purpose of the present study was to identify a CRC-specific vitamin D cut-off correlated with survival in patients with advanced CRC approaching a standard first-line chemotherapy. Moreover, we tried to identify potential determinants of vitamin D deficiency especially among immune-related variables. Finally, we also attempted to further refine survival in vitamin D deficient patients in order to generate suggestions on integrated targeted approaches for patient outcome improvement.

## 2. Materials and Methods

The patients under study described in the present paper were part of the population afferent to the study NCT01533740 and the REVERT trial (IRB approval n° 149.20 23 July 2020).

Three hundred forty-three patients with histologically confirmed CRC and measurable metastatic disease treated at the Tor Vergata University Hospital of Rome, Italy, with a standard first-line regimen between December 2010 and April 2021 were retrospectively evaluated. First-line regimens administered were either FOLFIRI (fluorouracil/irinotecan) or FOLFOX (fluorouracil/oxaliplatin) plus either an anti-epidermal growth factor (EGFR) agent (panitumumab or cetuximab) or an anti-vascular endothelial growth factor (VEGF) agent (bevacizumab) according to the RAS/BRAF mutational status (wild-type or mutant, respectively). Only patients whose plasma 25(OH)D was measured at baseline (within one week before treatment start) were included in the present analysis.

### 2.1. Optimal Vitamin D Deficiency Cut-Off Identification

First, we attempted to identify the optimal plasma 25(OH)D cut-off value predictive of overall survival (OS) in the study patients. For this purpose, a Hazard Ratio Smoothed curve with Restricted Cubic Splines (HRSC-RCS) [20] with 5 knots was initially used to unravel non-linear correlation between 25(OH)D and OS. Hazard Ratio curves represent the computation of pointwise estimates of the Hazard Ratio for increasing values of a continuous prognostic factor and unravel possible non-linear associations between the factor and the hazard. Then, a maximally selected rank statistics (MSRS) analysis was carried out to define the optimal cut-off value to predict poor survival in vitamin D deficient patients [21]. MSRS allows to identify the cut-point of a continuous variable with the optimal log-rank test when the variable is dichotomized to assess the impact on a survival outcome. Kaplan–Meier and Cox-regression analyses were performed to confirm the survival effect of plasma 25(OH)D, either by using the identified cut-off value or 25(OH)D as continuous variable. Given the importance of KRAS, NRAS and BRAF mutations for the outcome of mCRC patients, a subgroup analysis of the vitamin D survival effect according to the mutational status together with the interaction test was carried out as a meta-analysis and presented as a Forest plot.

After the cut-off value identification, an analysis of candidate predictors of vitamin D deficiency was performed. Thirty-three baseline epidemiologic and biochemical parameters were retrospectively collected: time since diagnosis, primary removal, sidedness, synchronous vs. metachronous metastasis onset, liver vs. not-liver metastasis, rat sarcoma virus (RAS)/BRAF status, Karnofsky Performance status (KPS), age, gender, body mass index (BMI), hemoglobin (g/dL) (Hb), platelets (m^3^/µL) (PLT), monocytes (m^3^/µL) (Mono), lymphocytes (m^3^/µL) (lymph), neutrophils (m^3^/µL) (Neu), lymph/white blood cells (WBC) (%), Neu/WBC (%), neutrophils-to-lymphocyte ratio (NLR), platelet-to-lymphocytes ratio (PLR), systemic inflammatory index (SII) (plt*neu/lymph), d-dimer (ng/mL) (DD), carcinoembryonic antigen (ng/mL) (CEA), CA 19.9 (U/mL), creatinine (mg/dL) (Cre), alanine aminotransferase (ALT) (U/L), aspartate aminotransferase (AST) (U/L), alkaline phosphatase (UI/L) (ALP), total bilirubin (mg/dL) (BilT), gamma glutamyl transferase (UI/L) (γGT), C-reactive protein (mg/L) (CRP), glycemia (mg/dL) (Glu), albumin (g/dL) (Alb) and lactate dehydrogenase (U/L) (LDH).

Among these, the continuous ones were first optimally dichotomized by means of ROC curve analysis with vitamin D deficiency obtained as classification variable before proceeding with a multivariable logistic regression model.

A multivariable logistic regression analysis with Least Absolute Shrinkage and Selection Operator (LASSO) was performed, including all the 33 covariates described above to identify the one with the highest predictive power for vitamin D deficiency and odds ratio (OR), with relevant confidence intervals being estimated [22]. Distribution of the identified predictor among vitamin D groups was assessed by means of chi square test and Mann–Whitney–Wilcoxon test. Additionally, the impact of the predictor on survival according to vitamin D strata was assessed by means of Kaplan–Meier and Cox-regression analyses.

### 2.2. Inflammatory/Immune System Status Evaluation

In order to assess the interplay between the immune cell system and vitamin D deficiency, nine peripheral blood immune variables were analyzed in a subset of patients: frequency of CD4+ among lymphocytes (CD4/lymph), CD4+ among WBC (CD4/WBC), CD8+ among lymphocytes (CD8/Lymph), CD8+ among WBC (CD8/WBC), CD4/CD8 ratio, B-lymphocytes among lymphocytes (B/lymph), B-lymphocytes among WBC (B/WBC), NK cells among lymphocytes (NK/lymph) and NK cells among WBC (NK/WBC).

The Mann–Whitney–Wilcoxon test was used to analyze for differences of immune variables between vitamin D deficient and non-deficient patients.

All analyses were performed with the R software v.4.0.3 and MedCalc software version 20.006. All tests were considered statistically significant for two tail *p* values < 0.05.

## 3. Results

### 3.1. Optimal Vitamin D Deficiency Cut-Off Identification

One hundred thirty-three patients were included in the study (60 males and 73 females). Median age was 64 years (range 30–84 years). A total of 47 patients had liver metastases, and 36 had no liver metastases. The median 25(OH)D level was 10.8 ng/mL (range 3–53.4 ng/mL), with 60 deficient (<10 ng/mL), 37 insufficient (10–20 ng/mL) and 36 within normal range (>20 ng/mL) levels, according to the standard cut-offs used for osteoporosis diagnosis [23] (Table S1). Patients were treated with first-line FOLFOX-panitumumab RAS/BRAF wild-type (56%) or FOLFOX-bevacizumab if RAS/BRAF mutated (44%). Ten percent of patients received 400 UI/day of vitamin D supplementation on a regular basis, with no apparent meaningful impact on plasma 25(OH)D levels.

**Figure 1:**
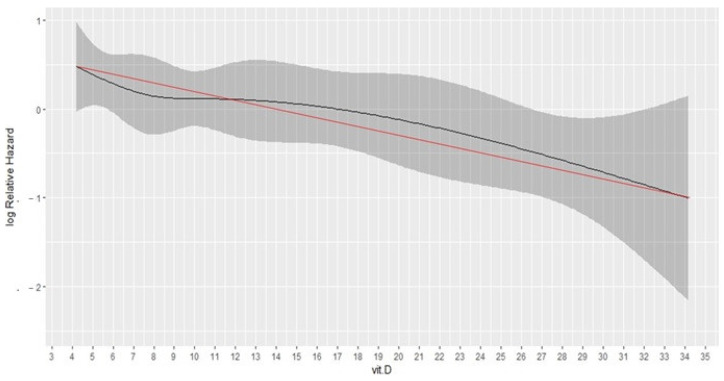
Hazard Ratio Smoothed Curve with Restricted Cubic Splines for the effect of continuous value of plasma 25(OH)D on overall survival. The graph demonstrates an approximately linear association with vitamin D (simplified by red straight-line). Vit.D: vitamin D; 25(OH)D: plasma 25-hydroxyvitamin D.

The HRSC-RCS curve revealed an approximately linear association between vitamin D values and risk of death (Figure 1). According to a Cox-regression analysis using plasma 25(OH)D as the continuous variable, 1 unit increase in 25(OH)D was associated to a 4% reduction in the risk of death (HR 0.96, 95%CI 0.93–0.99, *p* = 0.007).

The maximally selected rank test statistics was used to identify the most prognostic cut-off of 25(OH)D. We found that the cut-off of 10 ng/mL, that is also the cut-off universally used for osteoporosis, was the most significant (Figure S1).

**Figure 2:**
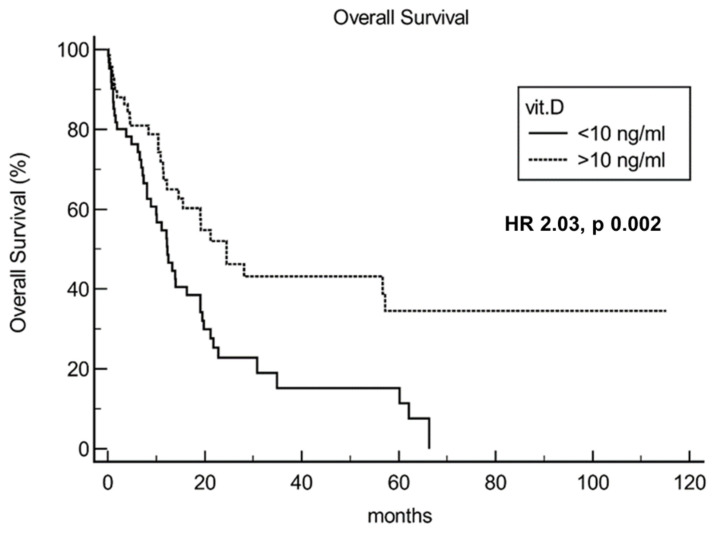
Survival analysis evidenced different survival according to vitamin level (< vs. >10 ng/dL). Vit.D: vitamin D.

mOS for 25(OH)D < 10 vs. > 10 ng/mL was 12.3 vs. 24.5 months, respectively, HR 2.03 [95% confidence interval (CI) 1.29 to 3.26], *p* = 0.002 (Figure 2).

**Figure 3:**
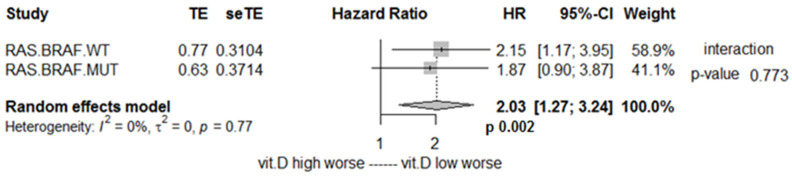
Impact of vitamin D on overall survival according to RAS and BRAF mutational status presented as meta-analysis. RAS.BRAF.WT: patients with RAS and BRAF wild-type. RAS.BRAF.MUT.: patients with either RAS or BRAF mutation; vit.D: Vitamin D.

No interaction was found between 25(OH)D and tumor mutational status for the effect on survival, with increased risk of death associated to vitamin D deficiency both in RAS/BRAF wild-type tumors (treated with anti-EGFR based first-line therapy) and in RAS or BRAF mutated tumors (treated with anti-VEGF based regimen), HR 2.15 and 1.87, respectively, *p* for interaction = 0.773 (Figure 3).

#### Vitamin D Deficiency Predictors

Thirty-three, clinical and hematochemical variables were investigated as potential predictors of Vitamin D deficiency in a multivariable logistic regression model.

Continuous covariates were conveniently dichotomized, using ROC curve analyses with vitamin D deficiency as classification factor (data not shown). Categories for the 33 covariates were therefore set as follows: time since diagnosis < vs. > 18 months, resection of the primary (yes vs. no), primary location rectum vs. colon, synchronous vs. metachronous metastasis onset, liver vs. not-liver metastasis, RAS/BRAF status (mutant vs. wild-type), KPS > vs. ≤ 80, age < vs. > 60 years, gender male vs. female, BMI < vs. > 20, Hb > vs. <11 g/dL, plt > vs. < 250 m^3^/µL, Mono > vs. < 0.6 m^3^/µL, lymph > vs. < 2 m^3^/µL, Neu > vs. < 5 m^3^/µL, lymph/WBC < vs. > 20%, Neu/WBC < vs. > 70%, NLR > vs. < 3.5, PLR > vs. < 250, SII > vs. < 1200, DD > vs. < 700 ng/mL, CEA > vs. < 15 ng/mL, CA19.9 > vs. < 15 U/mL, Cre > vs. < 0.6 mg/dL, ALT > vs. < 20 UI/L, AST > vs. < 15 UI/L, ALP > vs. < 80 UI/L, BilT > vs. < 0.7 mg/dL, γGT > vs. < 100 UI/L, CRP > vs. < 30 mg/L, Glu > vs. < 110 mg/dL, Alb > vs. < 3.5 g/dL and LDH > vs. < 200 U/L.

**Figure 4:**
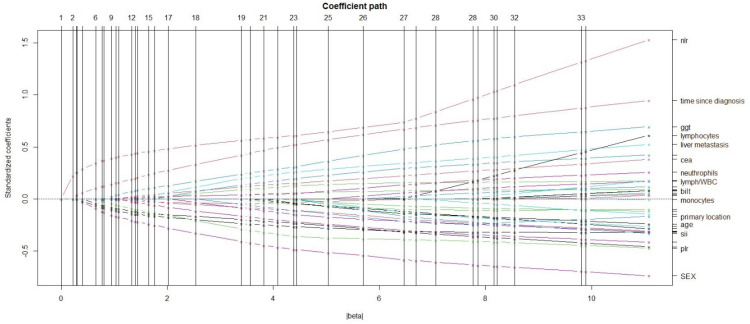
Path of coefficients for a multivariable logistic regression with Least Absolute Shrinkage and Selection Operator (LASSO) analysis of 33 candidate predictors. NLR was identified as the most powerful predictor of vitamin D deficiency.

At a multivariate logistic regression with LASSO, NLR > 3.5 was found to be the most powerful predictor of vitamin D deficiency with an OR of 3.35, 95% CI 1.63 to 6.92, *p* value = 0.0009 (Figure 4).

Proportion of NLR > 3.5 among vitamin D deficient vs. non deficient patients was 56% vs. 27%, chi-square *p* value = 0.0009 (Figure S2). The median NLR among vitamin D deficient and non-deficient patients was 3.6 vs. 2.9, respectively, Mann–Whitney–Wilcoxon test *p* value = 0.03.

**Figure 5:**
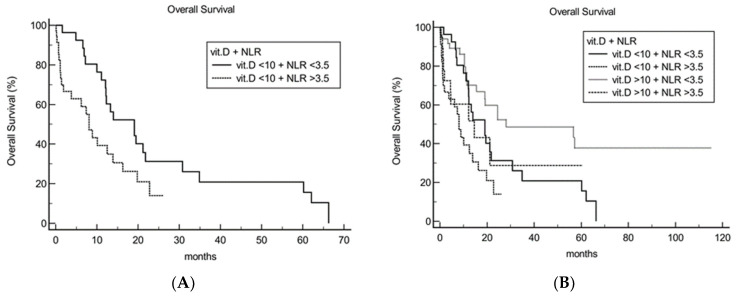
Survival of vitamin D low (25(OH)D <10 ng/mL) (**A**) and high (25(OH)D >10 ng/mL) (**B**) patients could be further stratified by NLR level (< vs. >3.5). NLR: Neutrophil-to-lymphocyte ratio; vit.D: Vitamin D.

NLR was also investigated as a prognostic factor in conjunction with 25(OH)D level. NLR was able to further stratify prognosis among patients with vitamin D deficiency: mOS in patients with 25(OH)D < 10 ng/mL for NLR < 3.5 vs. >3.5 was 19.1 vs. 8.1 months, respectively, HR 2.10 (95% CI 1.10 to 3.99), *p* = 0.02 (Figure 5A).

Survival analysis stratified by NLR and 25(OH)D level demonstrated that patients with NLR > 3.5 plus 25(OH)D < 10 ng/mL and patients with NLR < 3.5 plus 25(OH)D > 10 ng/mL had the shortest and longest mOS, respectively, 8.1 vs. 28.1 months, HR 3.40 (1.76–6.59), *p* = 0.0004 (Figure 5B).

### 3.2. Immune System Status Evaluation

Since NLR was found to be an important predictive factor of vitamin D deficiency, we investigated potential immune changes in peripheral blood between vitamin D deficient vs. non deficient patients, in a subset of patients with available circulating lymphocyte subpopulation frequency. CD4/lymph, CD4/WBC, CD8/lymph, CD8/WBC, CD4/CD8 ratio, B/lymph, B/WBC, NK/lymph, NK/WBC frequencies were available for 32 out of the 133 patients. The Mann–Whitney–Wilcoxon test was performed to compare the differences in the nine above reported variables between vitamin D deficient (<10 ng/dL) and non-deficient (>10 ng/dL) patients (Table 1). Deficient patients had statistically significant higher CD4+ T cells (median 48% vs. 40%, *p* 0.04) and lower B-lymphocytes (median 4% vs. 7%, *p* = 0.03) frequency among lymphocytes, as compared to non-deficient patients.

## 4. Discussion

In the present study, we analyzed the effect of plasma 25(OH)D levels on survival of metastatic colorectal cancer patients starting a standard first-line chemotherapy [24]. The prognostic value for vitamin D in cancer patients, including colorectal cancer, has been widely documented in recent years [19,25,26]. However, a mCRC-specific plasma 25(OH)D cut-off predictive of overall survival has not been investigated so far.

We found an approximately linear association between 25(OH)D and survival; however, the best cut-off value to predict prognosis was <10 ng/mL (25 nmol/L), which is remarkably close to the risk threshold for other skeletal and extra-skeletal conditions and has been unanimously recommended by international nutritional guidelines [27,28,29]. Importantly, the 10 ng/mL cut-off has been used as a reference value in most interventional studies regardless of other influencing factors such as age, sex, BMI and renal function. In our study, all these covariates were considered in the multivariable logistic regression model.

Notably, almost half of the patients (60 out of 133, 47.4%) in our cohort had a baseline plasma 25(OH)D level < 10 ng/mL and median level in the entire cohort was 10.8 ng/mL. This is in line with previously reported data in mCRC patients with median levels ranging from 10 to 18 ng/mL [15,29]. Tumor intrinsic features, such as KRAS, NRAS and BRAF mutational status, did not interact with the survival effect of 25(OH)D levels (*p* = 0.773) and this was in line with a recently published review [30]. A further analysis with a larger sample size taking into account the distinct subtypes of KRAS mutations would be of interest given a possible interaction with specific KRAS variants [31].

The optimal plasma 25(OH)D level that should be achieved is a matter of controversy, with many experts suggesting concentrations > 20 ng/mL (50 nmol/L) [14]. Given the high depletion observed in metastatic colorectal cancer patients, it is not surprising that vitamin D targeted therapy requires extensive doses of exogenous supplementation to demonstrate an impact on outcome in this setting.

In the recent SUNSHINE randomized trial, 139 mCRC patient candidates for a standard first-line chemotherapy were treated with mFOLFOX plus bevacizumab plus either standard dose of oral vitamin D3 (400 IU/d) or 10-fold higher dose of vitamin D3 (4000 IU/d). The high-dose group demonstrated a longer median progression-free survival as compared to the standard dose (13 vs. 11 months) but this was of borderline statistical significance according to the log-rank test (*p* = 0.07) [15].

Based on the pleiotropic effects of vitamin D, hypovitaminosis D has also been implicated in many other skeletal, cardiovascular, infectious, metabolic and pulmonary diseases. However, trials investigating the efficacy of targeted vitamin D3 supplementation at doses of 2000–4400 IU/d were not able to demonstrate an improvement in the outcomes in settings such as tuberculosis, asthma, diabetes and arterial thromboembolic events. [32,33,34,35].

The disappointing results obtained with targeted vitamin D supplementation might rely on the insufficient doses administered, especially if no stratification by baseline serum 25(OH)D is performed, or on the presence of concomitant factors that might have an influence in patients with vitamin D deficiency.

We scrutinized 33 common clinical and hematochemical variables, including those notably associated with 25(OH)D variations (i.e., BMI, age, creatinine, gender), and found that the well-known inflammatory index, neutrophil/lymphocyte ratio (NLR), displayed the most powerful association with vitamin D deficiency. Moreover, NLR significantly influenced the outcome of patients in conjunction with plasma 25(OH)D, as patients with 25(OH)D < 10 ng/mL plus NLR > 3.5 had a significantly shorter survival as compared to patients with 25(OH)D < 10 ng/mL plus NLR < 3.5 (*p* = 0.0004). Our results are in agreement with previously reported studies that correlated 25(OH)D with NLR in cardiovascular disease and diabetes [36,37,38,39].

Inflammation is notoriously involved in all phases of cancer growth, with a number of cytokines, chemokines and immune cells of the tumor microenvironment involved [39].

Moreover, systemic inflammation has consistently been reported as associated with decreased 25(OH)D concentrations. Furthermore, an anti-inflammatory property of exogenous vitamin D has been suggested [40,41].

Vitamin D as anti-inflammatory/immunomodulatory agent relies on the modulation of several pathways such as prostaglandin, nuclear factor kappa-light-chain-enhancer of activated B cells (NF-KB) and P38 mitogen-activated protein kinase (MAPK) [42,43]. It is exerted through its action on both innate and adaptive immunity since its receptor, VDR, is expressed and activated in many immune cells such as CD4 and CD8 T cells, B cells, neutrophils, macrophages, and dendritic cells [44]. It has been demonstrated that vitamin D silences CD4+ T helper 1 cells [45] and induces IL4, IL5, IL9 and IL13 release [46], which boost B cell proliferation and promote humoral immune system. Recent evidence has highlighted the role of vitamin D in restraining autoimmune diseases by reducing T helper 1 mediated response [45].

In vitamin D deficiency, an opposite scenario might be observed as evidenced in our study, with high CD4+ cells and low lymphocyte B [47,48,49,50]. The effect of these immune changes on patient prognosis is yet to be determined.

Hamada et al. evaluated the peri-tumoral lymphocyte reaction in conjunction with plasma 25(OH)D levels in more than 800 CRC cases. They observed that 25(OH)D significantly influenced cancer-specific risk of death only in tumors with low or null peri-tumoral lymphocyte reaction, thus reinforcing the hypothesis of an interaction between 25(OH)D levels and immune response [8]. In this study however, no specific analysis on B or T helper cells was performed.

Our results might provide suggestions on how to optimize future trials of vitamin D supplementation. We first encourage the design of specific trials for patients with plasma 25(OH)D < 10 ng/mL. Moreover, patient stratification according to baseline NLR would be desirable.

Patients with 25(OH)D < 10 ng/mL and NLR > 3.5 would possibly require ‘super-high’ vitamin D supplementation doses that have never been tested. Given the anti-inflammatory property of vitamin D, an extra-amount of exogenous vitamin D might be necessary to initially sedate inflammation in this setting of patients, before having an anticancer effect. Trials with very high doses of vitamin D (i.e., >4000 IU/d) should therefore be taken into consideration in our opinion.

Moreover, it would be of particular interest to investigate the effect of targeted combination treatment with vitamin D supplementation and immune-modulating agents in the setting of mCRC patients, especially in the case of hypovitaminosis D and high systemic inflammation.

Immunotherapy, namely anti-PD-1/PD-L1 antibodies, has extensively demonstrated efficacy in many solid tumors, but proved of limited success in colorectal cancer with both low vitamin D and high NLR being associated with inferior drug efficacy [51,52,53,54]. Since both exogenous vitamin D and anti-PD-1/PD-L1 agents might reduce systemic inflammation and favor antigen-specific anticancer immunity (as in the case of head and neck cancer patients [55]), we would encourage the design of trials investigating combination strategies of immunotherapy plus exogenous vitamin D.

Finally, a synergism has been observed with the combined use of corticosteroids, particularly dexamethasone, and vitamin D supplementation, for the beneficial impact on specific immune cells in diseases other than cancer (e.g., multiple sclerosis and asthma) [56,57]. It would be interesting to evaluate, in a randomized trial, the therapeutic effect of this combination in mCRC patients with hypovitaminosis D and high systemic inflammation.

We are acknowledging several limitations of our study. First, it is retrospective in nature and the sample size is limited. In addition, questionnaires on food intake were not administered to study patients; therefore, nutritional amount of vitamin D intake could not be considered. Only a minority of our patients were on regular oral vitamin D supplementation, and this did not change the overall final results.

Furthermore, it would be desirable to study a larger lymphocyte subpopulation analysis, also taking into consideration T and B-regulatory cells as well as the analysis of the complement system, given their possible role on vitamin D activation [45].

The dynamic evaluation of immune variables and plasma 25(OH)D during the course of the first-line treatment has not been performed. The longitudinal assessment of the variables would better define their interaction with the effect of the anticancer treatment and the evolution of the disease. The assessment of plasma 25(OH)D at different time-points (after 1 month, 3 months, 6 months and at disease progression of first-line) is currently underway in our center and we suggest including this longitudinal evaluation in future trials on vitamin D supplementation in metastatic cancer patients.

## 5. Conclusions

In conclusion, in the present study we present an mCRC-specific plasma 25(OH)D cut-off and implications of vitamin D deficiency on the immune system.

Collectively, these data suggest attempting an improvement of oral vitamin D targeted therapy either by further increasing the treatment doses, thus potentiating its anti-inflammatory properties (especially in patients with vitamin D deficiency and concomitant high systemic inflammation) or by integrating vitamin D supplementation with anti-inflammatory or immune-modulating agents.

## Figures and Tables

**Table 1:** Mann–Whitney–Wilcoxon test analysis of 9 immune/inflammatory variables frequencies among circulating lymphocyte subpopulation and among WBC in Vit D < and > 10 ng/mL paired cohorts (statistically significant values are evidenced in bold).

Parameter	Vit.D < 10 ng/dL	Vit.D > 10 ng/dL	*p* Value
	Median Values		
**CD4/lymph**	**48**%	**40**%	**0.04**
CD4/WBC	10%	9%	0.22
CD8/lymph	28%	27%	0.54
CD8/WBC	6%	6%	0.83
CD4/CD8 ratio	1.81	1.34	0.13
**B/lymph**	**4%**	**7%**	**0.03**
B/WBC	1%	1%	0.41
NK/lymph	16%	17%	0.82
NK/WBC	4%	3%	0.98

Vit.D: vitamin D; WBC: white blood cells; lymph: lymphocytes; NK: natural killer lymphocytes.

## Data Availability

The data presented in this study are available on request from the corresponding author. The data are not publicly available due to privacy regulations in force at our hospital.

## Supplementary Materials

The following supporting information can be downloaded at: https://www.mdpi.com/article/10.3390/cancers14112594/s1. Table S1: Patients’ characteristics; Figure S1: Maximally selected rank statistics analysis for overall survival defining the best cut-off value of vitamin D to stratify patients between good prognosis (longer survival) and poor prognosis (shorter survival); Figure S2: NLR < or >3.5 prevalence according to Vit.D levels.
