# Supplementary material for: Immune Response in Vitamin D Deficient Metastatic Colorectal Cancer Patients: A Player That Should Be Considered for Targeted Vitamin D Supplementation
Source: Cancers (Basel). 2022 May 24;14(11):2594. doi: 10.3390/cancers14112594 (PMC9179512; doi:10.3390/cancers14112594)
Supplement: Supplementary file 1 [file cancers-14-02594-s001.zip › cancers-1720734-supplementary.pdf]

Table S1. Patients' characteristics.

Parameter	n/median (%) [range]
Gender	
Male	60 (45)
Female	73 (55)
Age	64 [30-84]
Liver metastasis	71 (53)
RAS/RAF Wild Type	75 (56)
RAS mutated	47 (36)
BRAF mutated	11 (8)
Primary resected	83 (62.4)
Metastasitic disease	
Synchronous	90 (67.6)
Metachronous	43 (32.4)
First line mAb	
anti-VEGF	58 (43.6)
anti-EGFR	75 (56.4)

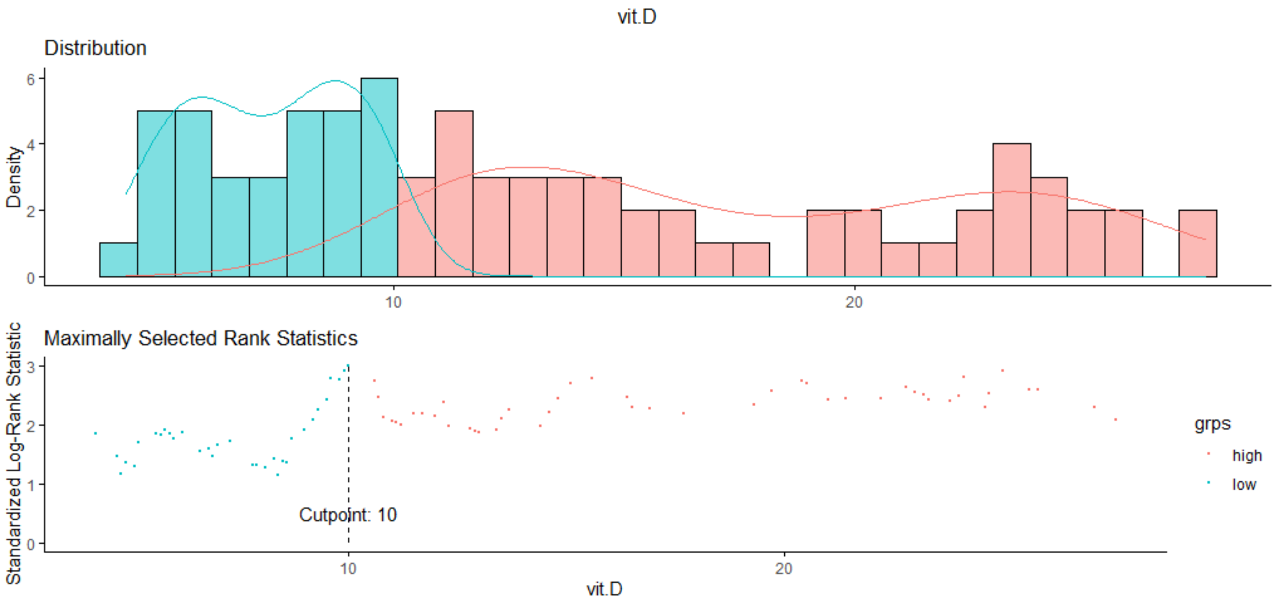

Figure S1. Maximally selected rank statistics analysis for overall survival defining the best cut-off value of vitamin D to stratify patients between good prognosis (longer survival) and poor prognosis (shorter survival). vit.D: vitamin D. Numbers refer to plasma 25(OH)D level in ng/mL. Best cutpoint: 10 ng/mL.

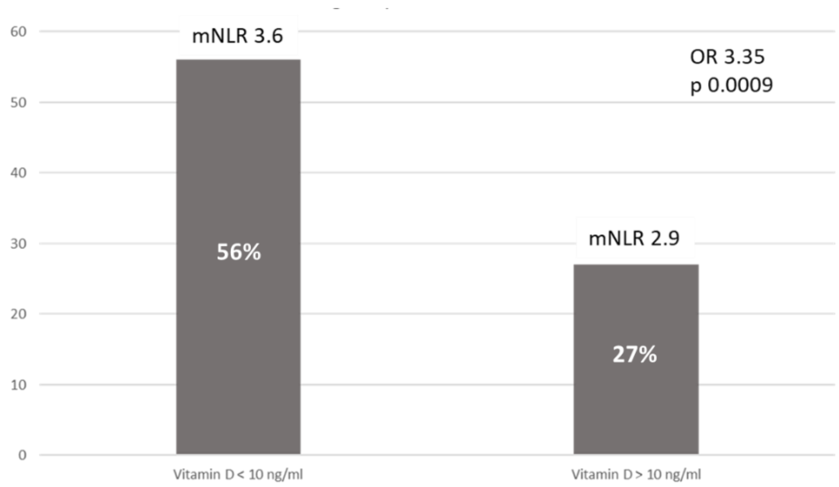

Figure S2. NLR < or > 3.5 prevalence according to Vit.D levels. NLR: neutrophils-to-lymphocytes ratio; Vit.D: vitamin D.
